# *Porphyromonas gingivalis* triggers the shedding of inflammatory endothelial microvesicles that act as autocrine effectors of endothelial dysfunction

**DOI:** 10.1038/s41598-020-58374-z

**Published:** 2020-02-04

**Authors:** Isaac Maximiliano Bugueno, Fatiha Zobairi El-Ghazouani, Fareeha Batool, Hanine El Itawi, Eduardo Anglès-Cano, Nadia Benkirane-Jessel, Florence Toti, Olivier Huck

**Affiliations:** 1 INSERM (French National Institute of Health and Medical Research), UMR 1260, Regenerative Nanomedicine, Fédération de Médecine Translationnelle de Strasbourg (FMTS), Strasbourg, France; 2 Université de Paris, Innovative Therapies in Haemostasis, INSERM UMR_S 1140, F-75006 Paris, France; 3 Université de Strasbourg, Faculté de Chirurgie-dentaire, 8 rue Sainte-Elisabeth, 67000 Strasbourg, France; 4 Faculté de Pharmacie, Université de Strasbourg, 8 rue Sainte-Elisabeth, 67000 Strasbourg, France

**Keywords:** Infection, Chronic inflammation

## Abstract

A link between periodontitis and atherothrombosis has been highlighted. The aim of this study was to determine the influence of *Porphyromonas gingivalis* on endothelial microvesicles (EMV_Pg_) shedding and their contribution to endothelial inflammation. Endothelial cells (EC) were infected with *P. gingivalis* (MOI = 100) for 24 h. EMV_Pg_ were isolated and their concentration was evaluated by prothrombinase assay. EMV_Pg_ were significantly increased in comparison with EMV_Ctrl_ shedded by unstimulated cells. While EMV_Ctrl_ from untreated EC had no effect, whereas, the proportion of apoptotic EC was increased by 30 nM EMV_Pg_ and viability was decreased down to 25%, a value elicited by *P. gingivalis* alone. Moreover, high concentration of EMV_Pg_ (30 nM) induced a pro-inflammatory and pro-oxidative cell response including up-regulation of TNF-α, IL-6 and IL-8 as well as an altered expression of iNOS and eNOS at both mRNA and protein level. An increase of VCAM-1 and ICAM-1 mRNA expression (4.5 folds and 3 folds respectively (*p* < 0.05 vs untreated) was also observed after EMV_Pg_ (30 nM) stimulation whereas *P. gingivalis* infection was less effective, suggesting a specific triggering by EMV_Pg_. Kinasome analysis demonstrated the specific effect induced by EMV_Pg_ on main pro-inflammatory pathways including JNK/AKT and STAT. EMV_Pg_ are effective pro-inflammatory effectors that may have detrimental effect on vascular homeostasis and should be considered as potential autocrine and paracrine effectors involved in the link between periodontitis and atherothrombosis.

## Introduction

Periodontitis is an infectious inflammatory disease associated with soft tissue inflammation and destruction of the tooth-supporting tissues characterized by increased periodontal pocket depth, gingival bleeding and clinical attachment loss. Such disease impairs oral health related quality of life and is considered the main cause of dental mobility and tooth loss^1^. Periodontitis affects a large proportion of the worldwide population and prevalence of its severe forms is estimated to be around 11% with a peak incidence around 38 years of age^2^. The development of periodontitis is associated with the establishment of a dysbiosis characterized by the predominance of anaerobic species, including *Porphyromonas gingivalis* (*P. gingivalis*), and an imbalanced host-immune response inducing periodontal tissue destruction^3^.

During the last decades, periodontal diseases were associated with various chronic diseases such as diabetes, rheumatoid arthritis, adverse pregnancy outcomes and cardiovascular diseases, suggesting a systemic impact^4–6^ with an enhanced proportion of worsened cardiovascular outcomes^7–9^. Indeed, patients with periodontitis are more prone to endothelial dysfunction, aneurysmal disease progression^10^, coronary artery narrowing, and an increased all-cause and cardio-vascular related mortality^11,12^. Moreover, in a clinical trial, intensive periodontal treatment of patients with severe periodontitis, improved at 6 months the endothelial dysfunction, a well-known marker of vascular injury^7^ thereby strengthening the causative link.

Among the proposed biological mechanisms^3,5,10,13^, the impact of oral and more specifically periodontal bacteria, on arterial homeostasis^14^ is suggested by their eventual dissemination in blood flow from the periodontal pocket. Interestingly, they were detected in atheromatous plaques as well as in the wall of healthy vessels in patients suffering from mild to severe periodontitis^15,16^. *P. gingivalis*, an anaerobic bacteria considered as a keystone periodontal pathogen, was demonstrated, *in vitro*^17–20^ and *in vivo*^21,22^ as a pro-inflammatory and pro-atheromatous mediator. Indeed, *P. gingivalis* exhibits a large number of virulence factors such as lipopolysaccharide (*Pg*-LPS), fimbriae or gingipaïns, contributing to the modulation of the innate immune response mainly through activation of Toll-Like-Receptors (TLRs)–dependent pathways^1,5,17,18^. Nevertheless, infection by *P. gingivalis* enhances endothelial inflammation or cell death in response to either low-density lipoprotein cholesterol (LDL) or pro-inflammatory cytokines (TNF-α), two circulating mediators associated with elevated cardiovascular risk, thus, highlighting a potential impact in the development of atherothrombosis^18^. However, the hypothesis of a detrimental effect solely induced by direct infection remains controversial as clinical trials assessing the preventative effect of antibiotic therapy did not show significant benefit in the secondary prevention of cardiovascular acute events in patients with history of myocardial infarction or myocardial ischemia^23^. Recently, the CANTOS trial pointed out inflammation as a key driver of atherothrombosis in patients with pro-inflammatory background. However, anti-interleukin-1β (IL-1β) antibodies although improving the cardiovascular outcomes failed to reduce cardiovascular mortality^24^. The eventual vascular impact of a variety of inflammatory mediators acting as autocrine or paracrine cellular effectors has been proposed^25,26^. Amongst them, microvesicles of endothelial origin (EMV) shed from the inflamed endothelium in response to infection could be the possible missing link between the infection-driven and the pro-thrombotic vascular responses.

Microvesicles (MV), also termed microparticles, are plasma membrane vesicles ranging from 50 nm to 1 µm released from stimulated cells. They contain a variety of active molecules such as lipids, enzymes, receptors and microRNAs. One characteristic feature of MV is that they expose phosphatidylserine (PhtdSer), an anionic phospholipid translocated from the inner to the outer leaflet of the plasma membrane. In addition, membrane proteins at the surface of the mother cell allow the characterization of their cell origin in body fluids. Circulating procoagulant EMV have been demonstrated as relevant biomarkers of vascular insult of atherothrombotic, inflammatory or mechanical origin, including ischemia reperfusion. Regardless of their cell origin, circulating MV emerge as the new actors of cellular crosstalk acting as procoagulant, pro-inflammatory, apoptotic or senescent pathogenic messengers under pathological conditions. Verily, the initial cellular stress at the origin of the MV shedding appears of relevance in the induction of a specific cell dysfunction^27^. In the context of infectious disease, the shedding of CD105^+^EMV has been proven to have a prognosis value in sepsis-induced coagulopathy^28^, whereas circulating MV of platelet and leukocyte origin released upon inflammation favor the recruitment of leukocytes at the surface of the inflamed endothelium^29,30^.

This study aims to determine *in vitro* the influence of *P. gingivalis* infection on EMV shedding (EMV_Pg_) and to evaluate an eventual autocrine action of EMV_Pg_ as noxious effectors possibly contributing to the dissemination of endothelial cell inflammatory responses and dysfunction.

## Materials and Methods

### Cell culture

Human umbilical vein endothelial cells (EC) (HUVEC, PromoCell, Heidelberg, Germany) were cultured in EGM2 medium (Promocell, Heidelberg, Germany) supplemented with 10% Fetal Bovine Serum at 37 °C in a humidified atmosphere with 5% CO_2_. Culture medium was changed each 3 days and no antibiotics were added to the medium.

### Endothelial cell infection by *P. gingivalis*

*P. gingivalis* strain 33277 (ATCC, Manassas, VA, USA) was cultured under strict anaerobic conditions at 37 °C in brain-heart infusion medium (Sigma-Aldrich, Saint-Quentin Fallavier, France) supplemented with hemin (5 μg/ml) and menadione (1 μg/ml) (Sigma-Aldrich). Bacteria were collected and counted as previously described^31^. Twenty-four hours before infection, 2 × 10^5^ EC/ml were seeded per well in a 24-well plate. EC were washed twice with PBS before infection with *P. gingivalis* at a multiplicity of infection (MOI) of 100. After 2 h of infection, medium was removed and infected EC were washed three times with PBS to remove non-adherent and external bacteria. Then, metronidazole (200 μg/mL) was added for 1 h to kill external bacteria and, after washing, 1 mL of fresh medium was added in each well. For comparative purposes, in some experiments, EC were stimulated with *P. gingivalis* ultrapure lipopolysaccharide (*Pg-*LPS) (1 μg/ml) (InvivoGen, San Diego, CA, USA) for 24 h.

### Measurement of EMV released in EC supernatant

After 24 h, supernatants from LPS-treated or *P. gingivalis*-infected EC were collected under sterile conditions. Detached EC and debris were discarded by low speed centrifugation (300 g; 15 min). EMV were then concentrated after two successive centrifugations (14000 g; 1 h at 4 °C), collected in 400 to 600 μl of HBSS (Hanks’ Balanced Salt solution) (Sigma-Aldrich) and stored at 4 °C. EMV concentration was measured by prothrombinase assay of the MV ubiquitous Phtdser exposure as previously described. Briefly, EMV were captured onto insolubilized Annexin-V, a protein with high affinity for Phtdser, using streptavidin-coated microtitration plates (Roche Diagnostics, Germany). After three washings, EMV were measured by prothrombinase assay in which blood clotting factors and calcium concentrations ensure that the PhtdSer borne by EMV is the rate-limiting parameter in the generation of soluble thrombin from prothrombin. After 10 min of incubation at 37 °C with human FXa (106 pM, Hyphen Biomed, France), Factor Va (FVa, 250 pM, Sekuisu, USA), prothrombin (FII, 3.5 µM, Hyphen BioMed, Paris, France) and CaCl_2_, thrombin generation was assessed in a multiplate spectrophotometer at 405 nm (Versamax, Molecular Devices, Wokingam Berkshire, UK) using a chromogenic thrombin substrate (PNAPEP0216 1.52 mM, Cryopep, Montpellier, France). Results were expressed as nanomolar PhtdSer equivalents (nM PhtdSer eq.) by reference to a standard curve established using liposomes of known composition and concentration^32^.

### Treatment of EC with EMV

EC were seeded in a 24-wells plate at 2 × 10^5^ cells/well. After 24 h, attached EC were washed and incubated with 5 to 30 nM of EMV_Ctrl_ (unstimulated EC) or EMV_Pg_ according to the experiment.

### Measurement of cell metabolic activity

Cell metabolic activity was determined using colorimetric AlamarBlue test (Life Technologies, Saint-Aubin, France) as described previously and according to manufacturer’s instructions^33^. Briefly, after 24 h of stimulation with EMV or *P. gingivalis*, 300 μl of cell supernatants were transferred to 96-well plates and optical density was measured at 570 and 595 nm in order to determine the percentage of AlamarBlue reduction.

### Determination of the cell viability

The cellular viability was evaluated using a fluorescence-based LIVE/DEAD® assay (LIVE/DEAD® Cell Imaging Kit, Molecular Probes™, Invitrogen) after 24 h of either infection or EMV stimulation. Cells were washed twice with PBS before staining. The staining solution consisted of 0.5 μL/mL calcein AM reagent and 2 μL/mL EthD-1 reagent mixed in 2 mL PBS. Samples were incubated for 10 min before analysis using a 10x and 20x objective lens of a fluorescence microscope (Olympus BX53F, Tokyo, Japan), filters for fluorescein and Texas Red for calcein and EthD-1 staining and digital CCD color imaging system (Microscope Digital Camera DP72; CellSens Entry®, Olympus, Tokyo, Japan).

### Measurement of cell apoptosis and necrosis

Annexin V-propidium iodide double staining was performed on washed trypsinized EC using the Annexin-V-FLUOS Staining Kit (Roche Applied Science, Meylan, France) according to manufacturer’s instructions. For each condition, a total of 1 × 10^4^ isolated cells were analyzed by flow cytometry using a BD^TM^ LSR II. The percentage of positive cells for IP^PE^ (necrosis), Annexin-V^FITC^ and IP^PE^ (late apoptosis), Annexin-V Positive Cells V^FITC^ (apoptosis), and unlabeled (viable cells) was determined from quadrant analysis.

### RNA isolation, reverse transcription and quantitative real-time PCR analysis

Total RNAs from samples were extracted using Tri reagent (Sigma-Aldrich) according to the manufacturer’s instructions. The total RNA concentration was quantified by spectrophotometry (NanoDrop 1000, Fischer Scientific, Illkirch, France) at 230 nm. Reverse transcription was performed using the iScript Reverse Transcription Supermix kit (Biorad, Miltry-Mory, France) according to the manufacturer’s instructions. qPCR was performed on the cDNA samples and gene expression was further analyzed using the CFX Connect^TM^ Real-Time PCR Detection System (Biorad, Miltry-Mory, France). Amplification reactions have been performed using iTAq Universal SYBR Green Supermix (Biorad, Miltry-Mory, France). β-actin was used as endogenous RNA control (housekeeping gene) in all samples. Primers related to β-actin, TNF-α, Il-6, Il-8, P21, P53, CDK4, eNOS, iNOS, SOD-1, VCAM-1, ICAM-1 and tissular factor (TF) were synthesized (ThermoFischer, Saint-Aubin, France) (Table 1). Expression level was calculated after normalization to the housekeeping gene expression.

**Table 1:** Primers sequences.

Gene (Human)	Sense strand	Sequences
β-actin	Forward Reverse	5′-AACGGCTCCGGCATGTGCAA-3′ 5′-CTTCTGACCCATGCCCACCA-3′
TNF-α	Forward Reverse	5′-AGGCGCTCCCCAAGAAGACA-3′ 5′-TCCTTGGCAAAACTGCACCT-3′
Il-6	Forward Reverse	5′-GCCTTCGGTCCAGTTGCCTT-3′ 5′-GCAGAATGAGATGAGTTGTC-3′
Il-8	Forward Reverse	5′-ATGACTTCCAAGCTGGCCGTGGCT-3′ 5′-TCTCAGCCCTCTTCAAAAACTTCTC-3′
P21	Forward Reverse	5′-TGGAGACTCTCAGGGTCGAAA-3′ 5′-GGCGTTTGGAGTGGTAGAAATC-3′
P53	Forward Reverse	5′-AACGGTACTCCGCCACC-3′ 5′-CGTGTCACCGTCGTGGA-3′
CDK4	Forward Reverse	5′-CATGTAGACCAGGACCTAAGG-3′ 5′-AACTGGCGCATCAGATCCTAG-3′
eNOS	Forward Reverse	5′-CGGCATCACCAGGAAGAAGA-3′ 5′-CATGAGCGAGGCGGAGAT-3′
iNOS	Forward Reverse	5′-TGGATGCAACCCCATTGTC-3′ 5′-CCCGCTGCCCCAGTTT-3′
SOD-1	Forward Reverse	5′-TAAAGTAGTCGCGGAGACGGG-3′ 5′-CGGCCTCGCAACACAAGCCT-3′
VCAM-1	Forward Reverse	5′-ATTGGGAAAAACAGAAAAGAG-3′ 5′-GGCAACATTGACATAAAGT-3′
ICAM-1	Forward Reverse	5′-GGCCGGCCAGCTTATACAC-3′ 5′-TAGACACTTGAGCTCGGGCA-3′
CD142 (TF)	Forward Reverse	5′-GACAATTTTGGAGTGGGAACCC-3′ 5′-CACTTTTGTTCCCACCTG-3′

### Proteome profile array of phospho-kinases

Total protein extraction and purification were performed from cell lysates as previously described^20^. Briefly, cells were lysed for 5 min on ice in 200 μl of ice-cold RIPA buffer (65 mM Tris–HCl, pH 7.4, 150 mM NaCl, and 0.5% sodium deoxycholate) supplemented with phosphatase inhibitor cocktails I and II and a protease inhibitor cocktail (Sigma, Darmstadt, Germany). Then, standardized concentration of total proteins was subjected to a Proteome Profiler Human Phospho-Kinase Array (R&D Systems, Lille, France) following manufacturer’s instructions. The density of spots, corresponding to protein activation, was measured by MyImage^TM^ Analysis Software 2.0 (Thermofisher) for each molecule and each condition.

### Western blotting

Briefly, cells were lysed for 5 min on ice in 200 μl of ice-cold RIPA buffer (65 mM Tris–HCl, pH 7.4, 150 mM NaCl, and 0.5% sodium deoxycholate) supplemented with phosphatase inhibitor cocktails I and II and a protease inhibitor cocktail (Sigma, Darmstadt, Germany). Lysates were centrifuged at 10,000 g at 4 °C for 10 min, supernatants were collected for quantification using the Bradford protein assay (Bio-Rad, Hercules, CA, USA) and 20 μg of cell lysates were loaded on a 12% SDS-PAGE for each condition. Antibodies against human eNOS (1/1000, mouse IgG, BD Biosciences, Le Pont de Claix, France), P21 (1/1000, rabbit IgG, Abcam, Paris, France), P53 (1/1000, rabbit IgG, Santa Cruz Biotechnology, Heidelberg, Germany), VCAM (1/500, rabbit IgG, Abcam, Paris, France), ICAM-1 (1/1000, mouse IgG, Life technologies, Courtaboeuf, France), CDK4 (1/1000, rabbit IgG, Life technologies), SOD-1 (1/1000, rabbit IgG, Life technologies), iNOS (1/1000, rabbit IgG, Life technologies) and against β-actin (1/2000, mouse IgG) from Santa Cruz Biotechnology (Heidelberg, Germany) were used for immunolabelling. Secondary alkaline phosphatase conjugated antibodies (anti-mouse (1/3000) or anti-rabbit (1/5000)) were purchased from Bethyl Laboratories (Montgomery, Texas, USA).

### Enzyme-linked immunosorbent assay

TNF-α secreted in the cell supernatant was assessed by a sandwich enzyme-linked immunosorbent assay (ELISA). Briefly, a goat anti-Human TNF-α antibody, (PeproTech, Rocky Hill, NJ, USA) was coated on a multi-well plate (4 μg/mL). Then, supernatants were added overnight at 4 °C. After washing with PBS, TNF-α was detected using a biotinylated goat anti-Human TNF-α (PeproTech). After 3 washing steps to discard unbound antibodies, HRP conjugated streptavidin (mix of solution A + B, as manufacturer instructions) was added and incubated for 20 to 30 minutes at room temperature. Finally, 100 μL of the stop solution (horseradish peroxidase and TMB substrate Solution, SS04, Life Technologies, Saint-Aubin, France) was added to each well for 5 to 30 min and then OD was measured by spectrophotometer (450 nm). The concentration of TNF-α was calculated by reference to a standard curve obtained with recombinant human TNF-α (PeproTech).

### Statistical analysis

Statistical analysis was performed using pair-wise Anova test and post-hoc Tukey’s test. Statistical significance level was considered for *p* < 0.05. Data were analyzed using PRISM 6.0 (GraphPad, La Jolla, CA, USA). All experiments have been performed at least three times from three different EC culture batches (biological and technical replicates).

## Results

### Endothelial microvesicles generated in response to *P. gingivalis* are pro-apoptotic endothelial effectors

**Figure 1:**
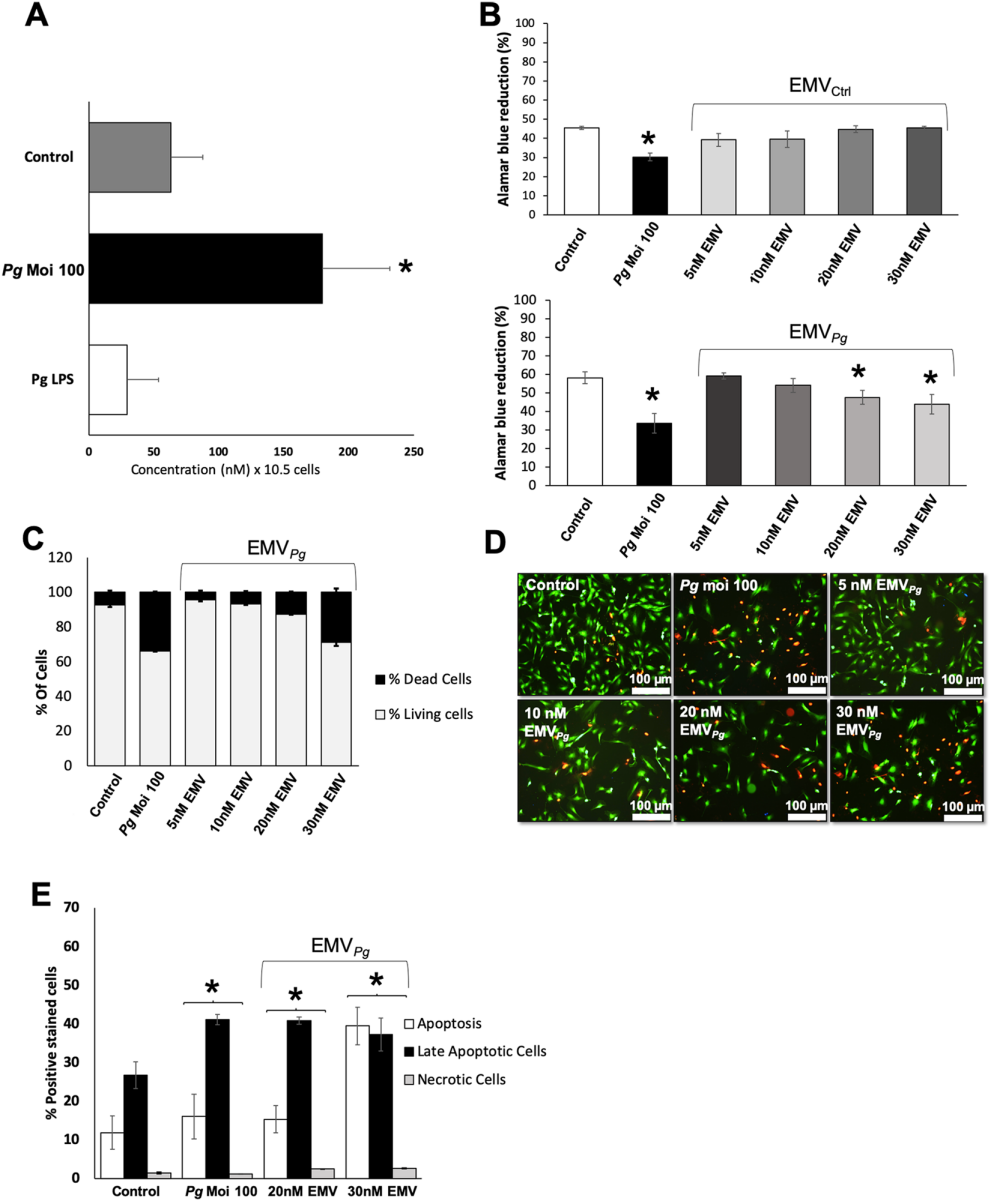
*P. gingivalis* promotes EMV shedding and alters endothelial cell viability. (**A**) The generation of EMV from naïve EC (control) and following 24 h of infection with *P. gingivalis* (*Pg)* (MOI = 100) or stimulation with *Pg*-LPS (1μg/ml) was measured in the supernatant by prothrombinase assay. Concentrations are represented by mean +/− SD from 3 independent experiments; *p < 0.05 vs control (unstimulated EC). (**B**) Metabolic activity of EC infected with *Pg* (MOI = 100) or exposed to EMV_Ctrl_ (upper panel) and EMV_Pg_ (lower panel) (5, 10, 20 and 30 nM) for 24 h measured by AlamarBlue assay. Results are expressed as mean +/− SD from 3 independent experiments; *p < 0.05 vs control (unstimulated EC). (**C**) Live-Dead assay to evaluate the ratio of live EC versus dead EC in cells exposed to *P. gingivalis* (*Pg*) (MOI:100) and EMV_Pg_ (5, 10, 20 and 30 nM) for 24 h. Results are expressed as percentage of live and dead EC (mean +/−SD). (**D**) Immunofluorescence imaging of live-dead staining (green: live EC, red: dead EC) for each condition after 24 hours of exposure. (**E**) Evaluation of type of cell death by flow cytometry after *P. gingivalis* infection and EMV_Pg_ (20 and 30 nM) exposure for 24 h. EC were labelled with Annexin-V FITC and propidium iodide (IPPE). All data were expressed as mean ± SD. *(*p* < 0.05 vs untreated cells).

*P. gingivalis* infection (MOI:100) led to a significant 2.8 fold in the shedding of EC after 24 h (infected *vs*. untreated, *p* < 0.05) (Fig. 1A) whereas the ultrapure *Pg*-LPS alone (1 μg/ml) had no significant effect (Fig. 1A). Interestingly, no MV shedding was detected in *P. gingivalis* culture supernatant (Supplementary Informations Fig. 1). The putative cytotoxic effect of EMV_Pg_ shed after *P. gingivalis* infection and of EMV_Ctrl_ shed from unstimulated cells was assessed after 24 h of incubation with the endothelial monolayer. While no cytotoxic effect has been observed for EMV_Ctrl_, increasing concentrations of EMV_Pg_ (5 to 30 nM) led to a significant 10% and 25% concentration-dependent reduction of cell viability and survival respectively (Fig. 1B–D, *p* < 0.05 vs untreated cells), values measured after 30 nM EMV_Pg_ treatment reaching the range of those elicited by *P. gingivalis* alone (MOI:100). A nearly 2-fold enhancement in the proportion of EC showing late apoptosis was observed in response to *P. gingivalis* infection or to 20–30 nM EMV_Pg_ by flow cytometry and fluorescence microscopy (*P. gingivalis*-triggered 1.7-fold fold *vs* untreated; 20 nM EMV_Pg_ -induced 1.6-fold *vs* untreated and 30 nM EMV_Pg_ -induced 1.5-fold *vs* untreated), while, the proportion of EC with early apoptosis was unchanged at 20 nM and drastically increased by 30 nM EMV_Pg_ suggesting a concentration threshold (Fig. 1E).

### EMV_Pg_ switch the mRNA and protein expression profiles of endothelial cells to a characteristic pro-inflammatory pattern

**Figure 2:**
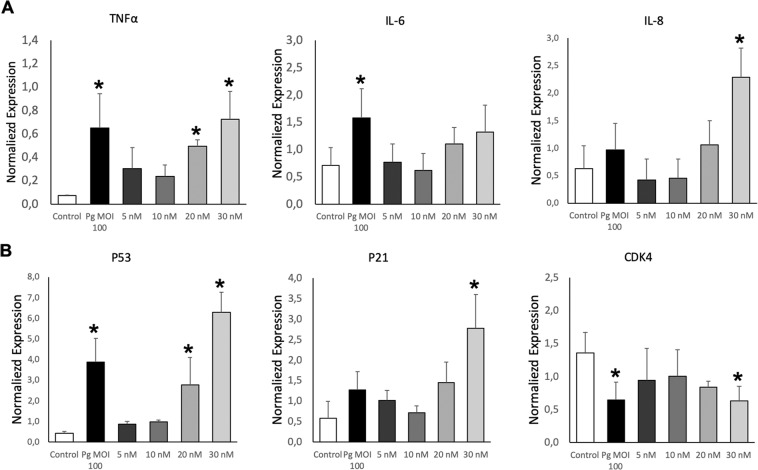
EMV_Pg_ trigger inflammatory endothelial response. (**A**) The mRNA expression of inflammatory markers TNF-α, IL-6 and IL-8 in EC exposed to *P. gingivalis* (*Pg*) (MOI:100) and EMV_Pg_ (5, 10, 20 and 30 nM) for 24 h was measured by qRT-PCR. (**B**) The gene expression of cell cyle related markers p53, p21 and CDK4 EC in EC exposed to *P. gingivalis* (*Pg*) (MOI:100) and EMV_Pg_ (5, 10, 20 and 30 nM) for 24 h. All data were expressed as mean ± SD from 3 independent experiments. *p < 0.05 between cells infected or stimulated against control (unstimulated cells).

**Figure 3:**
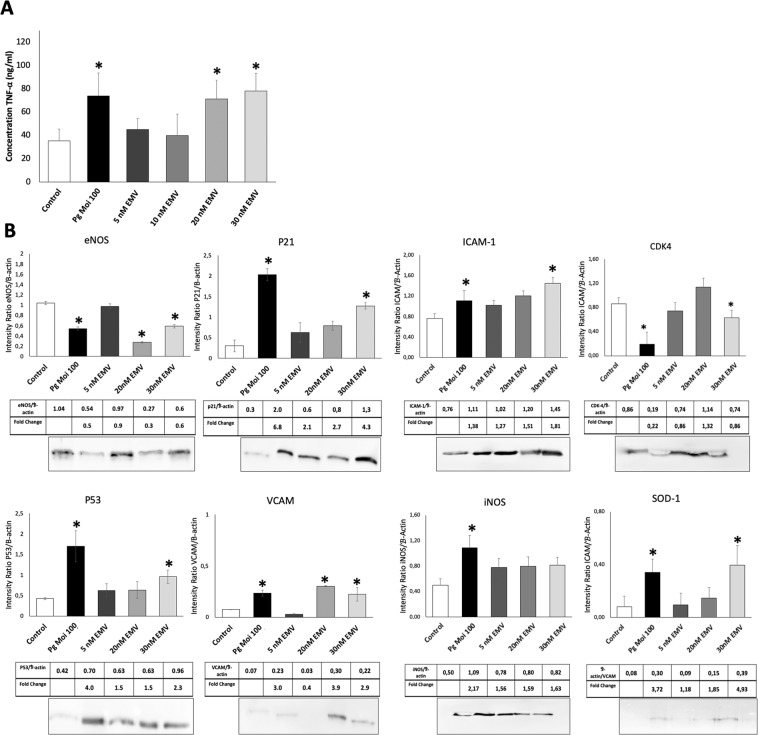
EMV_Pg_ induce pro-inflammatory and pro-oxidative protein expression similar to *P. gingivalis* infection. (**A**) TNF-α secretion in supernatant by EC in response to *P. gingivalis* (*Pg*) (MOI:100) and EMV_Pg_ (5, 20 and 30 nM) for 24 h was measured by ELISA. (**B**) Intra-cellular protein expression of eNOS, P21, ICAM-1, CDK4, P53, VCAM, iNOS and SOD-1 in response to *P. gingivalis* (*Pg*) (MOI:100) and EMV_Pg_ (5, 20 and 30 nM) for 24 h was evaluated by Western Blot. All data were expressed as mean ± SD from 3 independent experiments and normalized against internal control β-actin. *p < 0.05 between cells infected or stimulated against control (unstimulated cells).

**Figure 4:**
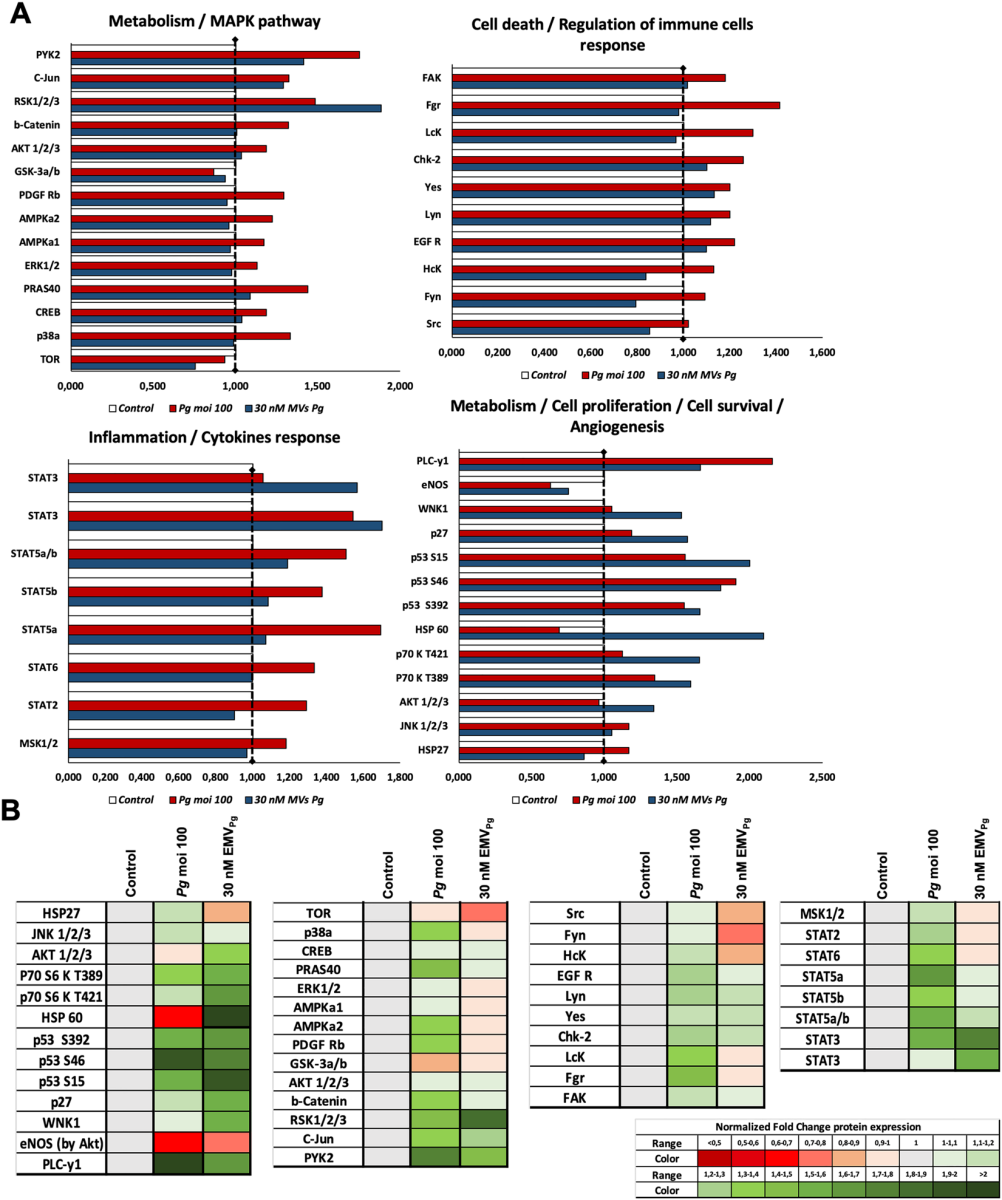
Exposure to EMV_Pg_ modulates significantly inflammatory pathways related to kinases activation. Analysis of kinases activation induced by *P. gingivalis* infection (*Pg*) (MOI:100) and EMV_Pg_ (30 nM) for 24 h evaluated by phospho-kinase array. The density of spots was measured by MyImage^TM^ Analysis Softwate 2.0 (Thermofisher) for each molecule and each condition. (**A**) Graphical representation of the kinases expression in untreated EC, in EC following *P. gingivalis* infection (*Pg*) (MOI:100) and EMV_Pg_ (30 nM) stimulation for 24 h. (**B**) Heat-map representation of the kinases expression normalized against untreated control (untreated EC).

The potential pro-inflammatory *P. gingivalis* or EMV_Pg_ -induced action was first assessed by the analysis of mRNA transcripts following a 24 h incubation with 5–30 nM EMV_Pg_. An up-regulation in the expression of pro-inflammatory TNF-α, IL-6 and IL-8 cytokines mRNA was detected, reaching similar TNF-α expression after either *P. gingivalis* infection or 30 nM EMV_Pg_ (6.5 folds *P. gingivalis* or EMV_Pg_ -induced *vs* untreated) (Fig. 2A). IL-6 mRNA expression was enhanced by 2.2 fold (*P. gingivalis* -induced *vs*. untreated; *p* < 0.05) whereas, IL-8 mRNA was highly and solely enhanced by 30 nM EMV_Pg_ by 6-fold. This pro-inflammatory switch was further confirmed by ELISA assay. Increased TNF-α concentrations after *P. gingivalis* infection (2.3 fold; *p* < 0.05) and EMV_Pg_ stimulation (2 folds; *p* < 0.05) were measured after 24 h of incubation (Fig. 3A). Interestingly, proteome analysis showed that several inflammation-related pathways are modulated following *P. gingivalis* or EMV_Pg_ stimulation (Fig. 4). Interestingly, not only did the JNK/AKT but also STAT were differentially up-regulated following endothelial infection or stimulation.

**Figure 5:**
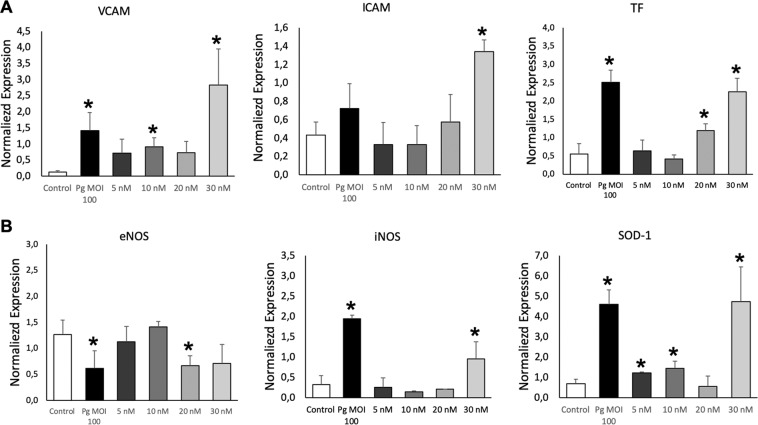
EMV_Pg_ induce expression of atherothrombosis and oxidative stress markers expression. (**A**) The mRNA expression of endothelial markers VCAM, ICAM and Tissular factor (TF) in EC exposed to *P. gingivalis* (*Pg*) (MOI:100) and EMV_Pg_ (5, 10, 20 and 30 nM) for 24 h was measured by qRT-PCR. (**B**) The gene expression of oxidative stress markers eNOS, iNOS and SOD-1 in EC exposed to *P. gingivalis* (*Pg*) (MOI:100) and EMV_Pg_ (5, 10, 20 and 30 nM) for 24 h. All data were expressed as mean ± SD from 3 independent experiments. *p < 0.05 between cells infected or stimulated against control (unstimulated cells).

Because leukocyte recruitment at the surface of the inflamed endothelium is one of the initial steps for atherothrombosis^34^, expression of endothelial VCAM-1 and ICAM-1 adhesion molecules that favor leukocyte attachment was also assessed. After 24 h incubation, 30 nM EMV_Pg_ significantly augmented both VCAM-1 and ICAM-1 mRNA expression (4.5 folds and 3 folds respectively (*p* < 0.05 vs untreated) whereas *P. gingivalis* infection was less effective at the chosen concentration, suggesting a specific triggering by EMV_Pg_ as compared to the above pro-inflammatory cytokines induction (Fig. 5A). Nevertheless, infection by *P. gingivalis*, significantly increased the expression of tissue factor (TF), the cellular initiator of the coagulation cascade at the endothelial surface, and also a pro-inflammatory and pro-apoptotic inducer^35^ after 24 h. Interestingly, EMV_Pg_ incubation led to a concentration-dependent expression of TF mRNA, reaching values prompted by *P. gingivalis* infection (Fig. 5A).

### EMV_Pg_ modulate endothelial oxidative stress and promote endothelial dysfunction

Because nitric oxide is one of the major vasoprotectors released by endothelial cells under physiological conditions and a very diffusible mediator of vaso-relaxation, we investigated whether the expression of NO synthases would be altered by EMV_Pg_. In this context, under conditions mimicking cardiovascular pathological conditions, like cytokine treatment or angiotensin II-induced endothelial dysfunction, iNOS, the inducible form of NO synthase (iNOS) is up-regulated, whereas, the constitutive eNOS expression remains unchanged^36^. Herein, 24 h after *P. gingivalis* infection or 20–30 nM EMV_Pg_ EC treatment, the expression of eNOS was significantly reduced at both transcriptional and protein levels (2.1 folds; *p* < 0.05) (Figs. 3B, 4, 5B). At contrary, increased iNOS expression was induced by *P. gingivalis* and EMV_Pg_ and enhanced by 2 and 5.8 folds respectively (*p* < 0.05 *vs*. untreated), thereby indicating endothelial dysfunction, eventually altered vascular function and possible eNOS uncoupling^37^. The expression of endothelial superoxide dismutase (SOD-1), an enzyme that rapidly inactivates superoxide anions, was also triggered by *P. gingivalis* and 30 nM EMV_Pg_ that both induced a 400% mRNA rise after 24 h, emphasizing the importance of cytoprotective signaling pathways in the endothelial response to major oxidative stress (Figs. 3B, 5B).

Because oxidative stress and the control of cell cycle are closely related, we also investigated whether *P. gingivalis* infection or EMV_Pg_ could also trigger regulators of the cell cycle. Indeed, both endothelial treatments targeted the oncosuppressor P53 and its downstream partner P21, a cyclin-dependent kinase inhibitor 1 and an inducer of senescence with a significant increase in P21 and P53 mRNA levels (4.7 and 14.3 folds respectively) (Fig. 2B). Moreover, the P53 up-regulation was confirmed by western-blot and proteomic analysis (Figs. 3B, 4). Conversely, a significant 2.3-fold decrease in the mRNA and protein expression of the cyclin-dependent kinase CDK4 was evidenced (*p* < 0.05 vs untreated) (Figs. 2B, 3B).

Altogether, these data strongly suggest that endothelial dysfunction in response to *P. gingivalis* infection or EMV_Pg_ stimulation is associated with oxidative stress and modulation of the cell cycle in a redox-dependent manner as reported in human and murine endothelial cells^36,38^.

## Discussion

This study demonstrates the autocrine pro-inflammatory properties of EMV_Pg_ shed from EC in response to *P. gingivalis* infection. Results indicate that after 24 h of incubation, the shed EMV_Pg_ appear as key mediators of endothelial dysfunction, switching the cytoprotective phenotype of endothelial cells toward a pro-inflammatory, pro-coagulant, pro-apoptotic and, eventually, a pro-senescent phenotype. All such phenotypes are a hallmark of sustained periodontitis in periodontal vascularization and atherothrombosis progression in large arteries. Furthermore, most of the cell responses investigated were of comparable severity whether initiated by *P. gingivalis* or by high concentration of EMV_Pg_.

Because MV are procoagulant owing to PhtdSer exposure and the eventual presence of TF when shed from monocytes, neutrophils and endothelial cells, their impact as pathogenic effectors in acute or chronic cardiovascular diseases and associated disorders such as myocardial infarction, atrial fibrillation, unstable angina, type-2 diabetes and their accumulation in the arteriosclerotic plaque has been extensively studied in relationship with the vascular inflammatory responses^27^. In blood, MV constitute a dynamic storage pool of vascular effectors, whereby, their cellular origin and concentration characterizing the severity of the disease or of organ damage. Of note, EMV, even circulating as a small proportion of the vascular pool were found of prognostic value in organ and cellular graft rejection^39^, pulmonary hypertension^40^, sepsis-induced coagulopathy^41^ and cardiovascular diseases^42–45^.

Strikingly, reports concerning infection-related MV shedding and how both host and pathogens produce MV are scarce. For instance, virulence factors, like LMP1 from the replicating Epstein-Barr virus, can be embedded in MV released from the infected cell and further spread virus through paracrine interactions with yet non-infected target cells. Similarly, human immunodeficiency virus also promotes MV shedding to transfer CCR5, its co-receptor on macrophages, to non-exposing target cells, consequently, promoting its own spreading^46^. MV hijacking by pathogens could also be the causative of link between periodontitis and cardiovascular disease, as strongly supported by our data and previous reports^47^. In congruence with our data, *Chlamydia pneumoniae* was also detected within atheromatous plaques and demonstrated to up-regulate the endothelial expression of the TF within 24 h together with the release of TF^+^-EMV that persisted for 1 week^48^. Most interestingly, we identified via proteomic and transcriptomic assays that the EMV shedding in response to *P. gingivalis* was associated with characteristic prerequisite pathways of the cytoskeleton proteolytic cleavage and plasma membrane remodeling preceding the MV release from the inflamed or stimulated endothelium^38^. Indeed, the P38 MAP kinase pathway that promotes the release of pro-inflammatory MV from human aortic endothelial cells in response to TNF-α^49^ was activated in our infection- driven model of pathogenic MV generation.

In this study, we emphasized the role of EMV_Pg_ as systemic pro-inflammatory and pro-oxidant effectors of endothelial responses mediated by *P. gingivalis*. In our *in vitro* MV-mediated model, EMV_Pg_ at 30 nM acted as cytotoxic endothelial effectors to an extent that was similar to the sole *P. gingivalis* infection (MOI = 100), via the activation of inflammatory pathways evidenced through Kinasome analysis. The up-regulation of TNF-α and IL-6 proinflammatory cytokines and their secretion eventually associated to an uncontrolled oxidative stress via the down-regulation of eNOS and the up-regulation of iNOS suggest NO synthase uncoupling. Therefore, our data altogether strengthen, the hypothesis of an EMV mediated *P. gingivalis* systemic response. This observation is strongly suggestive of a noxious role of EMV_Pg,_ eventually disseminated in blood flow to target healthy endothelial cells, even under conditions where its pathogenic inducer *P. gingivalis* would remain sequestered in the periodontal tissues by cells of the innate immune response^50^. Endothelial inflammation via TNF-α, IL-1β, or IL-6 pro-inflammatory cytokines favors leukocyte recruitment, endothelial dysfunction and activation of blood coagulation making interleukin-driven proatherothrombotic processes a potential pharmacological target explored in several clinical trials^51,52^. In view of the recent anti-interleukin therapy trials, that failed to demonstrate a benefit on cardiovascular outcome, circulating EMV released upon pathogen infection appear to be another target since EMV are able to concentrate at endothelial sites where flow disturbance favors enhanced interactions with MV, namely at artery branches prone to the development of atherotrombosis plaques. It was reported that MV sequestered in plaques act as paracrine endothelial up-regulators of pro-inflammatory ICAM-1 or VCAM-1 prompting leukocyte recruitment in the growing plaque^43,53^. Whether the plaque of patients subjected to chronic exposure of EMV released from distant endothelial infected sites undergo exaggerated endothelial inflammation remains to be demonstrated in specific animal models of atherothrombosis and in clinical trials. Nevertheless, we also showed that EMV_Pg_ triggered inflammation-related pathways. Indeed, the modulation of upstream NF-_K_B activators but also of transcription factors such as STAT confirmed their pro-inflammatory effect, as STAT pathway is a major signaling route converting the cytokine signal into gene expression programs regulating the proliferation and differentiation of the immune cell^54^.

*P. gingivalis* was previously demonstrated as a specific TLR4-dependent endothelial actor in pro-inflammatory TNF-α and IL-6 secretion following endothelial invasion or via its virulence factors such as LPS^55,56^. Moreover, several studies demonstrated the importance of NF-_K_B pathways in *P. gingivalis* elicited inflammation in several cell types^57–60^. In a previous report, we were able to demonstrate that TLR4^+^MV generated by *Pseudomonas* were true mediators of NF-_K_B-dependent signaling in TLR4^−^HEK engineered cells via I_K_B phosphorylation^61^. In the present study, our data confirm and extend these observations, by showing that EMV released upon *P. gingivalis* infection are autocrine endothelial effectors possibly contributing to plaque endothelium inflammation and erosion^62^. Such effect appears specific to the EMV shedding trigger as only EMV_Pg_ were able to induce a significant endothelial cell response while none was measured with EMV_Ctrl_. This observation highlights the need of deep analysis of the EMV_Pg_ content, including mRNA, miRNA and proteins, as it was shown that such content is highly influenced by both triggers; the cellular environment and pre-existing conditions^63,64^. It also paves the way of future clinical trials aiming to determine MV phenotype in the context of variable periodontitis severity and *P. gingivalis* infection.

Accumulation of ROS and endothelial redox imbalance have been widely demonstrated in the atherothrombotic plaque *in vitro*, in animal models and in human vascular tissues^43,65^. Oxidative stress is up-regulated upon infection by *Escherichia coli* or *Salmonella typhimurium*^66–68^. Conversely, *in vitro* models indicate that eNOS endothelial activity is reduced upon TNF-α or LPS incubation^69^ whereas experimental bacterial meningitis in engineered mice with iNOS invalidation support the hypothesis of a dual endothelial iNOS and eNOS up-regulation upon comparison with wild type individuals^70^. Here, EMV_Pg_ triggered oxidative stress via altered the expression of iNOS and eNOS at mRNA and protein levels, data altogether supporting eNOS uncoupling owing to major ROS accumulation and consecutive endothelial dysfunction, together with blunted cytoprotective signaling pathways against excessive inflammatory and pro-thrombotic responses. Interestingly, circulating MV in patients with coronary artery disease are elevated in comparison to that with healthy individuals and contain reduced functional eNOS. Furthermore, it was also demonstrated that a proportion of EMV circulating in such patients reduce the availability of NO in coronary artery endothelial cells and down regulate its expression^71^. Further studies are required to characterize a specific sub-population of endothelial MV eventually responsible for the systemic dissemination of the pathogen-driven inflammatory and pro-oxidant signals to uninfected endothelial cells and possibly contributing to endothelial dysfunction in patients with cardiovascular disease.

In this study, *P. gingivalis* strain ATCC 33277 was used as a pathogenic model. It should be mentioned that this strain is not the most virulent and future studies should determine the pro-inflammatory impact of others strains or mutants lacking fimbriae or gingipaïns as it was demonstrated a significant impact of such virulence factors on experimental outcomes in different cell types^72,73^. Future studies should also determine the precise composition of EMV_Pg_ as their content is clearly influenced by the quantity and type of cellular stress trigger. Indeed, a specific insight should be made on mRNA, miRNA, proteins and membrane receptors^64,74,75^.

In conclusion, *P. gingivalis*-induced EMV are effective pro-inflammatory effectors potentially involved in cardiovascular disease worsening. Nevertheless, autocrine and paracrine actions of EMV need further characterization of embedded noxious content, and of their cellular and subcellular molecular partners. Further studies are needed to understand the impact of sustained yet low bacterial or virulence factors as well as low grade cytokine dissemination. A specific emphasis should be done on more relevant *in vitro* models such as human aortic endothelial cells and *in vivo*. Causative links between periodontitis and cardiovascular diseases should be investigated to decipher the systemic routes of the periodontal pathogenic signal dissemination. Local EMV released at distance from the thrombogenic plaque are a good candidate.

## Supplementary information

Supplementary informations.
