# Supplementary material for: Porphyromonas gingivalis triggers the shedding of inflammatory endothelial microvesicles that act as autocrine effectors of endothelial dysfunction
Source: Sci Rep. 2020 Feb 4;10:1778. doi: 10.1038/s41598-020-58374-z (PMC7000667; doi:10.1038/s41598-020-58374-z)
Supplement: Supplementary file 1 — Supplementary informations. [file 41598_2020_58374_MOESM1_ESM.pdf]

***Porphyromonas gingivalis* triggers the shedding of inflammatory endothelial microvesicles that act as autocrine effectors of endothelial dysfunction**

Isaac Maximiliano Bugueno, Fatiha Zobairi El-Ghazouani, Fareeha Batool, Hanine El Itawi, Eduardo Anglès-Cano, Nadia Benkirane-Jessel, Florence Toti and Olivier Huck

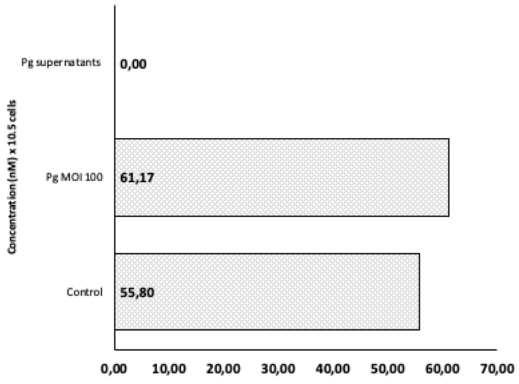

Supplemental Figure 1. EMV dosage. The generation of EMV from naïve EC (control), after 24h and of EC infected with *P.gingivalis* (*Pg*) (MOI=100) or *Pg* culture were measured in the supernatant by prothrombinase assay.

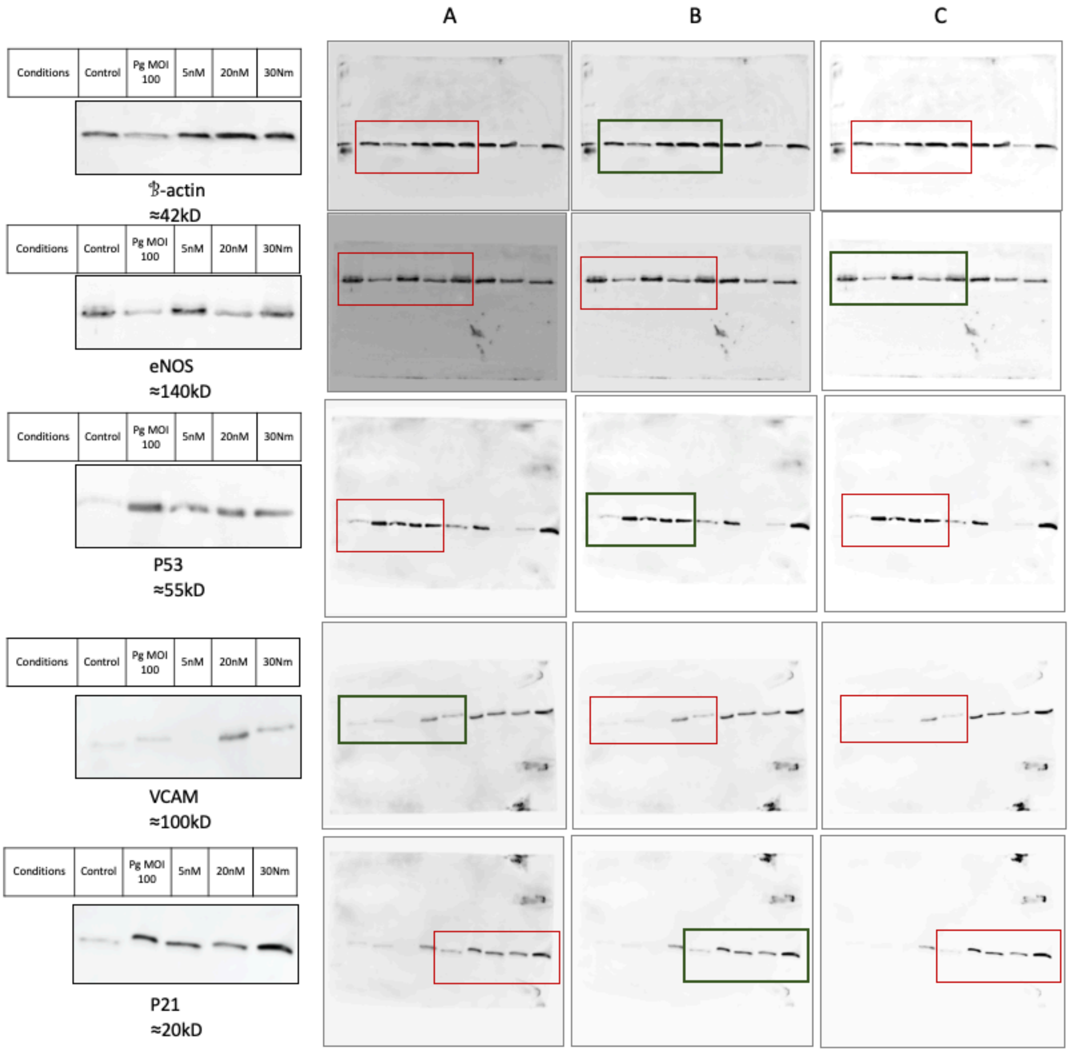

Supplemental Figure 2. Western Blots. The figure shows the original blots for ß-actin, eNOS, P53, VCAM and P21, that were cut to indicate only the conditions that were chosen (Control, *Pg*, EMV$_{Pg}$ 5nM, 20nM and 30 nM). Brightness and contrast modifications were performed as shown in panels A, B and C. The green boxes show the area that was presented in the left panel of the figure, the red boxes indicate the same area but with a different time exposure.

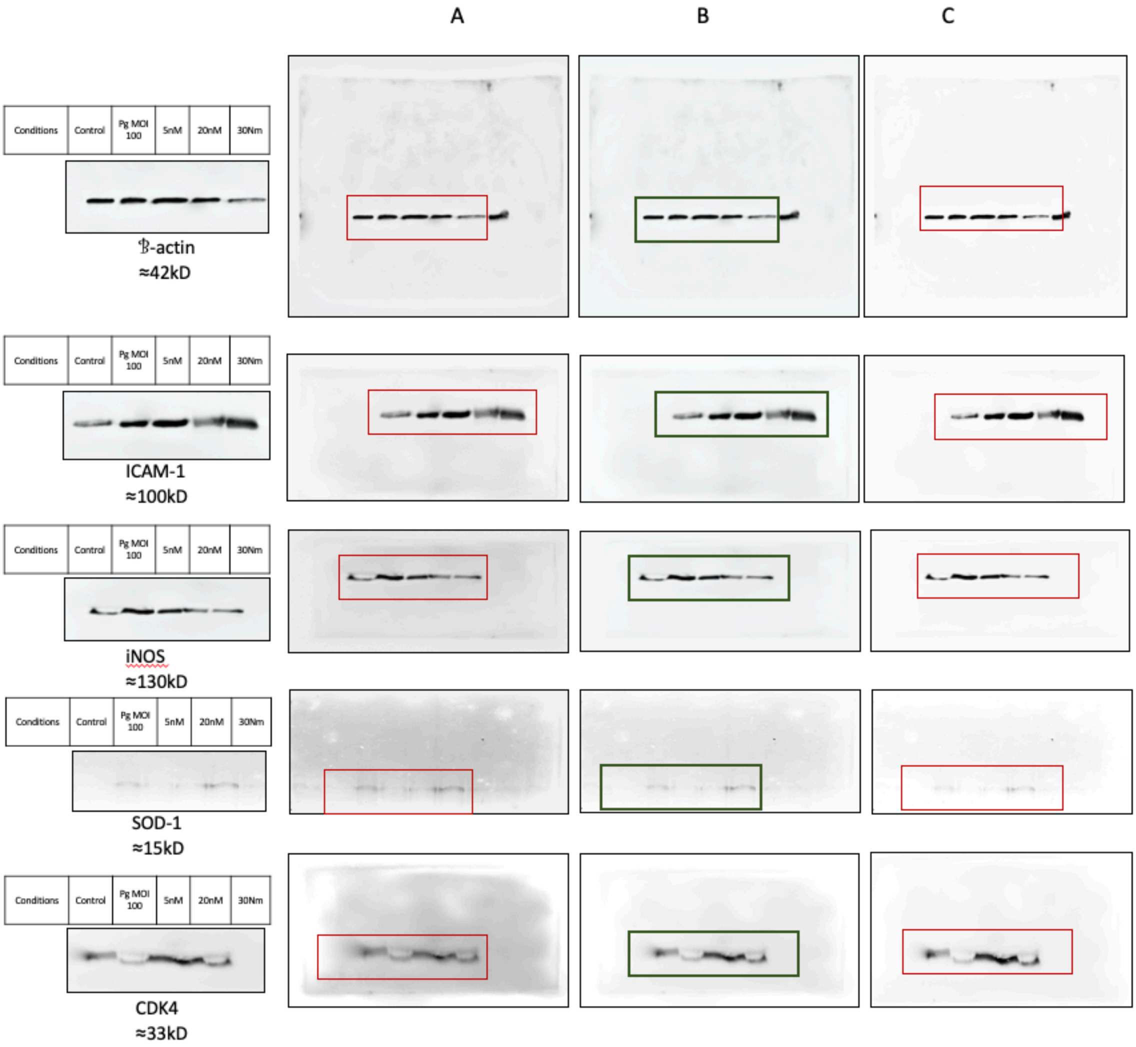

Supplemental Figure 3. Western blots. The figure shows the original blots for ϐ-actin, ICAM-1, iNOS, SOD-1 and CDK-4, that were cut to indicate only the conditions that were chosen (Control, *Pg*, EMV$_{Pg}$ 5nM, 20nM and 30 nM). Brightness and contrast modifications were performed as shown in panels A, B and C. The green boxes show the area that was presented in the left panel of the figure, the red boxes indicate the same area but with a different time exposure.

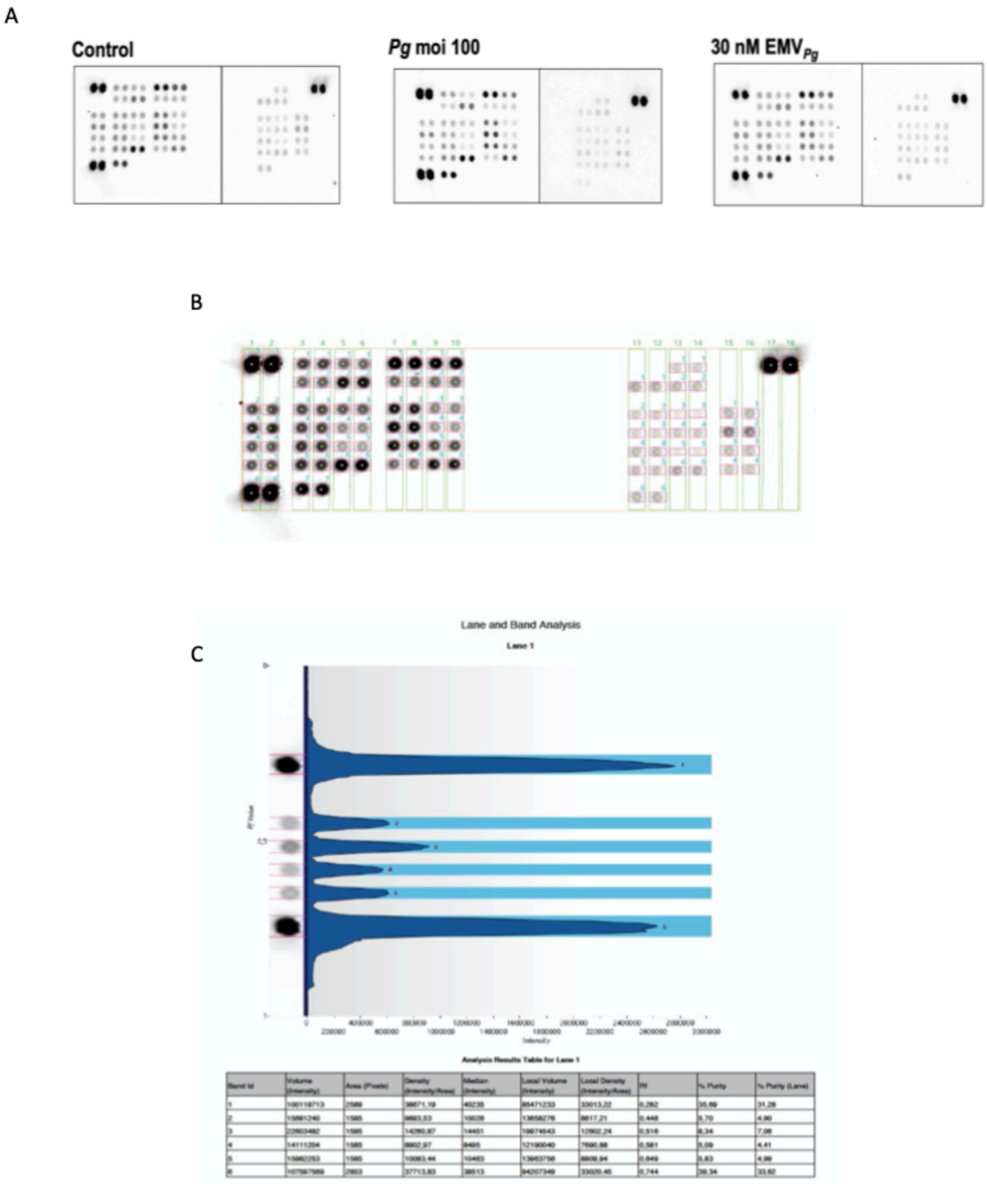

Supplemental Figure 4. Analysis of kinases activation induced by P.gingivalis infection (Pg) (MOI:100) and EMV$_{Pg}$ (30 nM) for 24 h evaluated by phospho-kinase array. (A) The density of spots was measured by MyImage^{TM} Analysis Software 2.0 (Thermofisher) for each molecule and each condition. (B-C) The figure shows how the analysis has been performed for each of the blots of the "Proteome Profiler Human Phopho-Kinase Array Assay" (R&D Systems, Lille, France). Each spot per column and per line was analyzed according to its intensity. This density of spots, corresponding to protein activation, was measured my MyImage^{TM} Analysis Software 2.0 (Thermofisher) for each molecule and each condition.
